# A theranostic 3D ultrasound imaging system for high resolution image-guided therapy

**DOI:** 10.7150/thno.71221

**Published:** 2022-06-27

**Authors:** Hanna Bendjador, Josquin Foiret, Robert Wodnicki, Douglas N Stephens, Zoe Krut, Eun-Yeong Park, Zulma Gazit, Dan Gazit, Gadi Pelled, Katherine W Ferrara

**Affiliations:** 1 Stanford University, Stanford CA, USA.; 2 University of Southern California, Los Angeles CA, USA.; 3 University of California, Davis CA USA.; 4 Cedars-Sinai Medical Center, Los Angeles, CA 90048, USA.

**Keywords:** Volumetric ultrasound imaging, microbubble imaging, image-guided therapy, CPS imaging, array design

## Abstract

Microbubble contrast agents are a diagnostic tool with broad clinical impact and an increasing number of indications. Many therapeutic applications have also been identified. Yet, technologies for ultrasound guidance of microbubble-mediated therapy are limited. In particular, arrays that are capable of implementing and imaging microbubble-based therapy in three dimensions in real-time are lacking. We propose a system to perform and monitor microbubble-based therapy, capable of volumetric imaging over a large field-of-view. To propel the promise of the theranostic treatment strategies forward, we have designed and tested a unique array and system for 3D ultrasound guidance of microbubble-based therapeutic protocols based on the frequency, temporal and spatial requirements.

**Methods:** Four 256-channel plane wave scanners (Verasonics, Inc, WA, USA) were combined to control a 1024-element planar array with 1.3 and 2.5 MHz therapeutic and imaging transmissions, respectively. A transducer aperture of ~40×15 mm was selected and Field II was applied to evaluate the point spread function. *In vitro* experiments were performed on commercial and custom phantoms to assess the spatial resolution, image contrast and microbubble-enhanced imaging capabilities.

**Results:** We found that a 2D array configuration with 64 elements separated by λ-pitch in azimuth and 16 elements separated by 1.5λ-pitch in elevation ensured the required flexibility. This design, of 41.6 mm × 16 mm, thus provided both an extended field-of-view, up to 11 cm x 6 cm at 10 cm depth and steering of ±18° in azimuth and ±12° in elevation. At a depth of 16 cm in B-mode, we achieved a volume acquisition rate of 60 Hz and an imaging display rate of 30 Hz. Lateral resolution was 0.8 mm at 5 cm depth and 2.1 mm at 12.5 cm depth.

**Conclusion:** A single 2D array for both imaging and therapeutics, integrated with a 1024 channel scanner can guide microbubble-based therapy in volumetric regions of interest.

## Introduction

The clinical interest in ultrasound-based approaches for imaging and therapy has been growing exponentially in the past thirty years. In biomedical imaging, the low cost, portability, deep-penetration, and lack of ionizing radiation have enhanced the utility of ultrasound as compared to magnetic resonance imaging (MRI) or x-ray techniques. In therapy, it is the potential to non-invasively target a small area without ionizing radiation that has enhanced clinical use 1. From tumor ablation with high-intensity focused ultrasound (HIFU) 2 to lithotripsy 1, therapeutic ultrasound techniques have proven their clinical efficacy. Thus, developing the technology for image-based therapy has the potential for significant theranostic impact. However, there is an unmet need for transducers and imaging protocols that facilitate volumetric ultrasound imaging and microbubble-based or low intensity focused ultrasound therapies.

Ultrasound imaging is undergoing a global and unprecedented transformation with the growth of 3D spatial 3-5, and 4D spatio-temporal imaging 6,7. Therefore, 2D arrays of various geometries have been developed, including 1.5D arrays 8-11 and 1.75D arrays 12 which provide elevational focusing. Sparse arrays were later developed with periodic or aperiodic element spacing to reduce the number of required channels 13,14. Row-column 15-18 geometries also addressed this issue by reducing the number of interconnections between the elements. Instead of addressing each element individually, the elements of a row-column array (RCA) are addressed by the index either of their row or of their column. An RCA can thus transmit with all columns, or all rows, at once and receive in either direction. This notably reduces both the hardware and computational costs but imposes significant limitations due to data sparsity, speckle noise and the spatially variant point spread function. 2D arrays 19 have also been developed for volumetric imaging using a micro-beamformer approach 20 to reduce the effective element count relative to the channel count of the system. This efficient approach can reduce an element count of ~9000 to a channel count of 128 or 256 channels. However, due to the use of purpose-designed and proprietary application specific integrated circuits (ASICs) microelectronics, open-source control of the scanning protocol for research of new imaging modalities is limited. Micro-beamformers are, in effect, closed systems where academics cannot reprogram the micro-beamformer, and therefore the ability for university researchers to develop flexible theranostic applications using these devices is reduced. Further, the micro-beamformer approach (with high voltage pulsers embedded in the ASIC in the handheld probe enclosure) may be limited in the transmitted power and burst length as well as waveform format and linearity when compared with a high-channel system where the pulsers are not contained within the transducer itself. Finally, due to system-level optimization in beamforming configurations for power and circuit complexity in micro-beamformer based probes, the flexibility to select arbitrary beam formation parameters is limited to a subset of possible strategies.

Ultrasound imaging guidance of ultrasound therapy has typically been conducted by connecting a therapeutic array with a 1D ultrasound imaging array, which is itself connected to a separate imaging system. Multiple approaches have been used to combine and co-register the separate imaging and therapeutic arrays 21-23. In some cases, the therapeutic array was capable of beam steering in 3D; however, imaging was limited to a single plane. A colinear array approach using two outer arrays with a total of 128 elements at 1.5 MHz and an inner imaging array with a higher center frequency (3-7 MHz) has been used to combine imaging and therapy; however, volumetric therapy was not feasible 24,25. Sparse hemispherical arrays have also been proposed for simultaneous ultrasound therapy and 3D microbubble monitoring under MRI guidance 26-33. However, the sparse element spacing does not allow for conventional ultrasound imaging and the imaging frequencies of these systems are below 1.5 MHz. These approaches thus rely on MRI guidance, which has limited the adoption of such therapies.

To the best of our knowledge, an array capable of ultrasonic anatomical imaging with the bandwidth and volumetric beamforming strategies required to realize both imaging and therapy with a single array has not been reported previously. Here, we create a flexible scheme in which a 1024-element array is interfaced to a 1024-channel system, providing the ability to optimize and test algorithms for imaging and therapy. Combining therapeutic and diagnostic modalities into one single system to enhance the treatment efficiency, a cornerstone of theranostics, led us to propose a single ultrasound array for 3D ultrasound imaging and 3D image-guided therapy. The development of ultrafast imaging has further contributed to this development. When using plane or diverging waves, the entire medium is insonified with a single transmission, and backscattered echoes are recorded from all elements of the array simultaneously. With fully-addressed 2D arrays, a 3D imaging volume can thus be rapidly accessed 4,34,35.

Microbubbles can be applied to enhance both the imaging and therapeutic applications of ultrasound. First, they can be used as contrast agents. In contrast-enhanced ultrasonic imaging (CEUS) 36-38, microbubble expansion and collapse increase near resonance or with decreasing transmission frequency. A center frequency of ~250 kHz to 1.5 MHz has been shown to facilitate cell transfection 39,40, and monitoring such treatment requires a wide bandwidth to assay harmonic spectral components. For image guidance, a higher transmission frequency, ideally greater than 2 MHz, is desirable to improve spatial resolution. Microbubbles are combined with ultrafast imaging in ultrasound localization microscopy (ULM) to achieve spatial resolution on the order of microns 41,42. When co-injected with or loaded with drugs or genes, microbubbles also enhance delivery and transfection 43-45. For instance, 1.3 MHz sonication with a 1.6 Mechanical Index (MI) facilitated efficient gene delivery to pancreatic cells 46. More recently, a similar protocol resulted in *in situ* mini-pig tibial bone regeneration 47. In general, a peak negative pressure on the order of hundreds of kPa is required 40. These challenging applications of ultrasound-mediated gene delivery would benefit from the ability to deliver and monitor the ultrasound treatment in 3D.

A further application of the technological need for microbubble imaging is 3D passive acoustic mapping 23,26-28, where the acoustic emissions from microbubbles are received passively. The microbubbles are insonified from a separate transducer or subaperture and the array is used to receive and map the microbubble echoes. With this technique, the insonified microbubble positions and cavitation regime can be determined 48-50. The lack of access to 2D arrays that can adequately localize the microbubble signal has limited progress in this area.

In summary, our goal is to integrate a 2D ultrasound transducer with a 1024 channel real time system to simultaneously perform 3D imaging at 2.5 MHz and image-guided therapy at 1.3 MHz over a range of target depths. We propose a flexible theranostic array that can be applied in a wide range of microbubble and low intensity focused ultrasound applications. We first present the clinical and practical motivations behind the array design and investigate its intrinsic acoustic and imaging properties. We then demonstrate *in vivo* human 3D imaging and the *ex vivo* guidance of therapeutic protocols.

## Array design and characterization

### Temporal requirements

For any given study, the dose of microbubbles is limited as well as the circulation time. Ideally, a single vial is injected per study. Independent of the rate of infusion, the optimal therapeutic strategy relies upon insonation of each microbubble as it flows through the region of interest (and that region is often small). In order to accomplish this optimal strategy, as large a fraction of the region as possible should be insonified instantaneously. In addition to performing therapy, our system also aims to monitor treatment in real time and in three dimensions with B-mode and contrast imaging. It thus needs to be able to acquire, transfer and process data rapidly. Ultrasound plane waves are ideal in this context since they insonify a larger region and can achieve a volume rate of 30 or more volumes per second 51. With the matrix array designed here and integrated with 1024 channels of system pulsers, each element is driven independently, and the entire array can transmit and receive simultaneously to achieve diverging waves and cylindrical or spherical beams. The 1024-channel system is composed of four synchronized Vantage 256 systems (Verasonics, Kirkland, WA) assembled together 52, each controlled by a graphics processing unit (GPU)-embedded computer. The direct integration of the 1024 elements with dedicated individual pulsers as opposed to a multiplexed scheme allows for the higher duty cycles that are required for most microbubble-based therapeutic protocols.

Fast acquisition is important for multiple reasons when guiding microbubble-based therapy: 1) the operator can move the transducer or inject microbubbles and needs real-time guidance, 2) patient movement must be monitored to adjust the localization of the therapy, 3) evaluation of the reflow (refresh) of microbubbles into the field-of-view occurs within seconds and quantification of the refresh rate requires a volume rate of 10 Hz or more. In order to characterize the temporal performance, we consider the volume rate and the image display rate. The *volume rate* is defined as the rate at which a volume can be acquired. It is determined by the pulse repetition frequency (PRF) (which is typically fixed by the imaging depth) and the number of transmissions (plane waves) required. We note that in the Verasonics 1024-channel configuration, the operating system overhead currently limits the PRF to ~4 kHz. The *image display rate* characterizes the rate at which the image refreshes, and is determined by volume acquisition rate, data transfer time, the processing (beamforming) time, and the time for display. We seek to 1) acquire at least 60 B-mode (or 20 contrast mode) volumes per second to follow cardiac and microbubble dynamics and 2) to display B-mode volumes at a minimum of 30 images per second based on the above reasoning.

### Spatial design requirements

Clinical applications spanning regenerative medicine and tumor therapies require depths from 3 to 10 cm and a field-of-view of centimeters in any dimension surrounding a lesion that is smaller than the field-of-view. When designing the array, applications envisioned included musculoskeletal lesions (e.g. non-healing fractures) and breast or abdominal tumors. According to 53, a useful approximation for estimating the limit on the depth of penetration in centimeters is given by 60/(frequency in MHz) which in this case yields 24 cm. We particularly focus on lesions 5 cm or less in each dimension and target therapeutic and imaging depths from 3 to 10 cm such that the imaging dynamic range remains in the typical 50-70 dB range. Both the azimuthal (long array dimension) and elevational (short array dimension) resolution are important for 3D imaging. Given anatomical constraints in this application, the required elevational aperture was limited to 16 mm to enable imaging between the ribs, and the designed azimuthal aperture (~40 mm) was chosen to facilitate treatment of shallow targets while still achieving high in-plane azimuth resolution imaging. This results in an open beamforming architecture 2D array with an aperture similar to the Philips X6-1 54,55 while avoiding the limitations of a fixed-design, proprietary micro-beamformer and embedded multiplexors and pulsers on ASICs. Our Verasonics based 1024-channel ultrasound system directly drives a two-dimensional array, and we aimed at independently driving each element. The target element count for the array was thus fixed at 1024, leading to the specifications in Table 1.

Given the limitation of 1024 simultaneous imaging channels, three configurations are feasible: 128 × 8 elements, 64 × 16 elements, and 32 × 32 elements. Considering the dimension specifications of ~40 mm in azimuth and ~15 mm in elevation (to allow for intercostal spacing), the pitch size corresponding to those three configurations was determined. In order to sweep the beam over a volume, the array must be designed such that grating lobes are acceptable for imaging and minimal in the therapeutic mode. To address both issues, the impact of pitch and element count on spatial resolution and grating lobes was evaluated using the Field II simulation toolbox for MATLAB 56,57. The three possible configurations were tested as follows:

- (128 × 8) 128 azimuthal elements with 0.5 λ-pitch and 8 elevational elements with 3 λ-pitch;
- (64 × 16) 64 azimuthal elements with λ-pitch and 16 elevational elements with 1.5 λ-pitch;
- (32 × 32) 32 azimuthal elements with 2 λ-pitch and 32 elevational elements with 0.75 λ-pitch, where λ is the imaging wavelength (0.62 mm with a speed of sound of 1540 m/s in tissue and 2.5 MHz imaging frequency), each generating an array aperture of ~40 mm in azimuth and ~15 mm in elevation.

We simulated the azimuthal and elevational dimensions as independent linear arrays. The “azimuthal” array describes one line of the prototype design, and the “elevational” array, one column. We first assessed the imaging capabilities of the array at a depth of 10 cm. We placed a reflecting point at 10 cm in the simulation and evaluated the conventional line-per-line pulse-echo response at 2.5 MHz with a 50% bandwidth. This corresponds to transmitting focused beams with an equal number of elements and transmissions. The resulting point spread functions (PSFs) were compared for the three sets of pitch values.

### Point spread function

**Figure 1:**
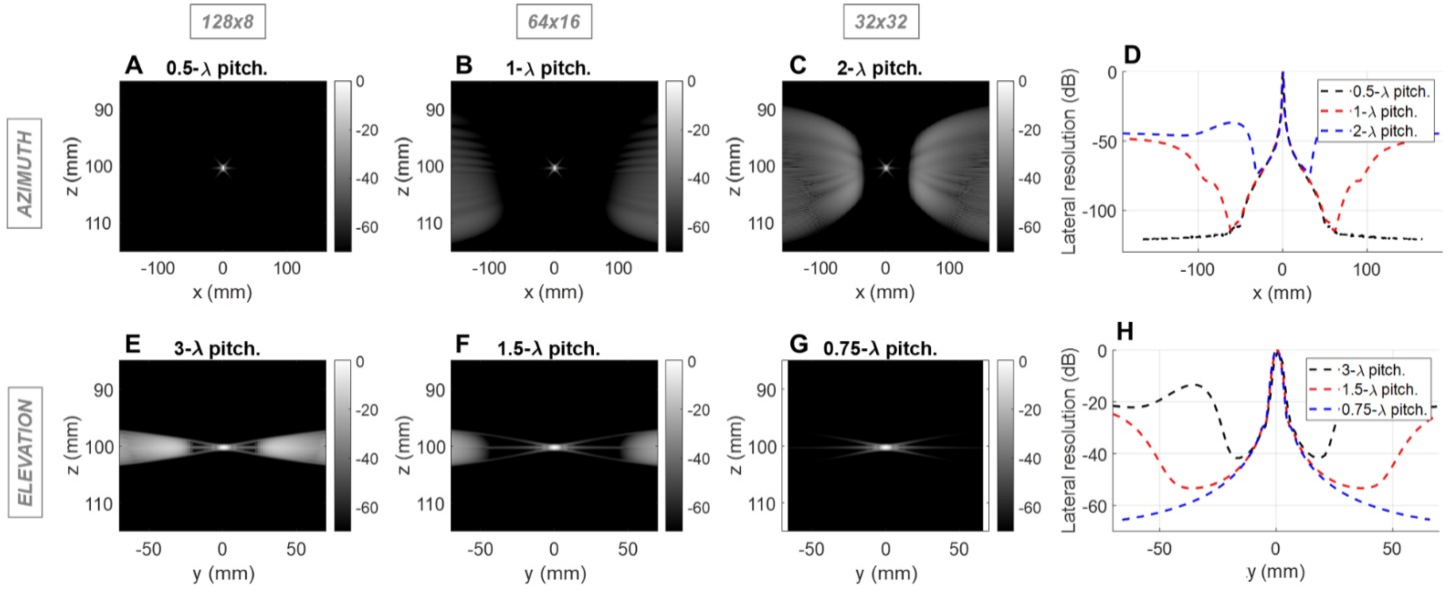
** Field II simulations of 2.5 MHz pulse-echo (2-way) point spread functions (PSFs) at 10 cm depth for three array configurations with constant aperture size.** Simulations of the azimuthal array: B-mode images for (**A**) 0.5λ - pitch array with 128 elements, (**B**) λ pitch array with 64 elements, and (**C**) 2λ - pitch with 32 elements; (**D**) Lateral resolution (cross-sections) of the PSFs. Simulation of the elevational array: B-mode images for (**E**) 3λ - pitch array with 8 elements, (**F**) 1.5λ - pitch array with 16 elements, and (**G**) 0.75λ - pitch with 32 elements, (**H**) Lateral resolution of the PSFs.

**Figure 2:**
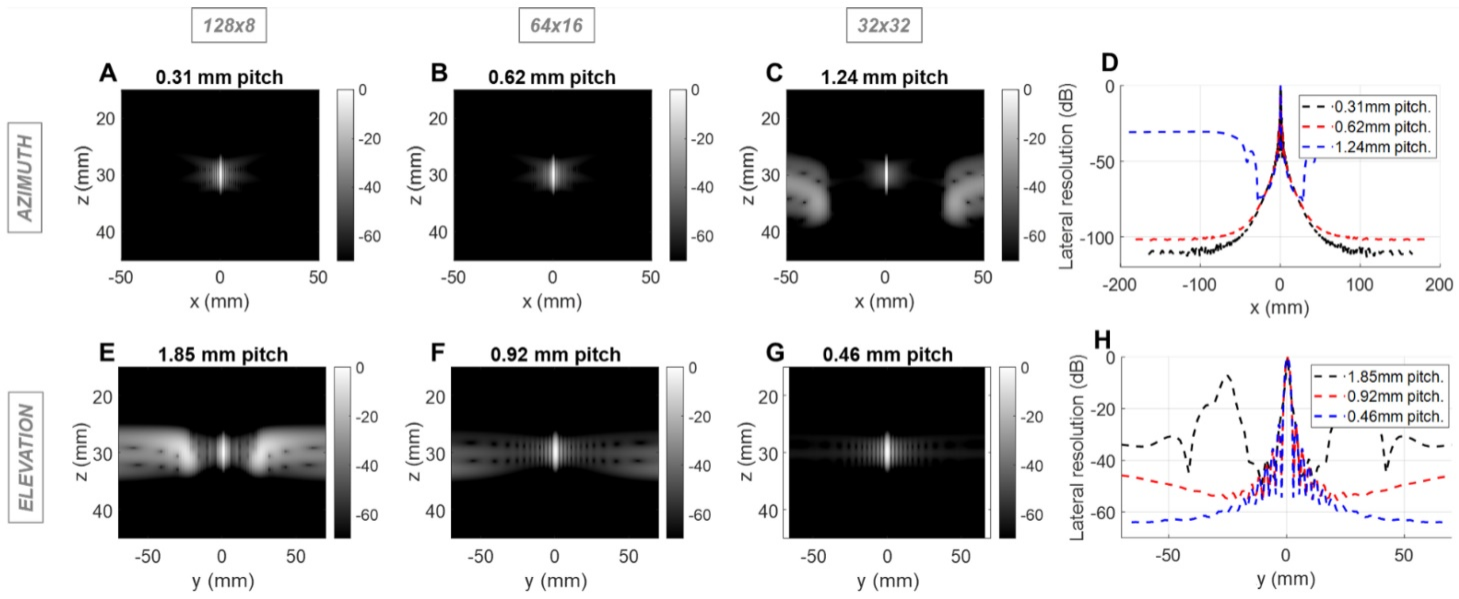
** Field II simulations of 1.3 MHz transmitted (1-way) point spread functions (PSFs) at 3 cm depth for three array configurations with constant aperture size.** Simulations of the azimuthal array: PSF intensities for (**A**) 0.31mm pitch array with 128 elements, (**B**) 0.62mm pitch array with 64 elements, and (**C**) 1.24mm pitch with 32 elements, (**D**) Lateral resolution of the PSFs. Simulation of the elevational array: PSF intensities for (**E**) 1.85 mm pitch array with 8 elements, (**F**) 0.92 mm pitch array with 16 elements, and (**G**) 0.46 mm pitch with 32 elements; (**H**) Lateral resolution of the PSFs.

For the imaging center frequency of 2.5 MHz at a depth of 10 cm, the -6 dB 2-way azimuthal PSF width was 1.4, 1.4 and 1.3 mm, respectively for the 128 × 8, 64 × 16 and 32 × 32 configurations and was ~3.9 mm in elevation for each configuration (Figure 1). The PSF was narrower for the 3 cm depth as expected (Figure S1). For therapeutic operation at 1.3 MHz, the -6 dB 1-way PSF was 0.8, 0.7 and 0.7 mm in azimuth and 2.0, 2.0 and 2.1 mm in elevation, respectively for the three configurations (Figure 2). As expected, the pitch impacts the lateral resolution once the aperture size has been fixed.

### Grating lobes

In the imaging configuration, for both the azimuthal and the elevational arrays, grating lobes appear only when exceeding 0.5 λ-pitch (Figure 1D) and approach the main lobe as the pitch increases. With the three proposed scenarios, the grating lobes appear in the under-sampled dimension in each case (elevation for 128 x 8, both dimensions for 64 x 16 and azimuth for 32 x 32). For the 128 × 8 array, the nearest grating lobe in elevation is -13 dB within 30 mm from the center of the array (Figure 1E). For the 32 × 32 array in azimuth, the grating lobe height is -36 dB within 60 mm (Figure 1C). For the 64 × 16 array, the grating lobe height remains lower than the 32 × 32 array in azimuth, and the 128 × 8 array in elevation and remains below -40 dB for a region of ±50 mm in either dimension (Figure 1B,1F). Thus, considering the typical image dynamics and the size of the region of interest, a configuration of 64 elements with a λ pitch in azimuth and 16 elements with a 1.5 λ-pitch in elevation would likely minimize the impact of grating lobes on the resulting image.

We next evaluated the effect of the pitch on the therapeutic performance for the same three configurations using Field II and Matlab. For therapy, the designed center frequency was 1.3 MHz where λ was ~1.2 mm. Simulation parameters include a 10% bandwidth and a depth of 3 (Figure 2) or 10 cm (Figure S2). We probed the one-way PSF of the transmitted beam. For clarity, we expressed the pitch in millimeters rather than in multiples of wavelength. Because the pitch is smaller than the therapeutic wavelength, grating lobes are reduced in height and shifted in location. Here, the improved performance of the 64 × 16 array is apparent in that the off-target intensity is below -40 dB over a range of positions spanning more than 60 mm in each dimension (Figure 2B, 2F). For the alternative designs, off-target intensity exceeded 40 dB within 25 mm.

**Figure 3:**
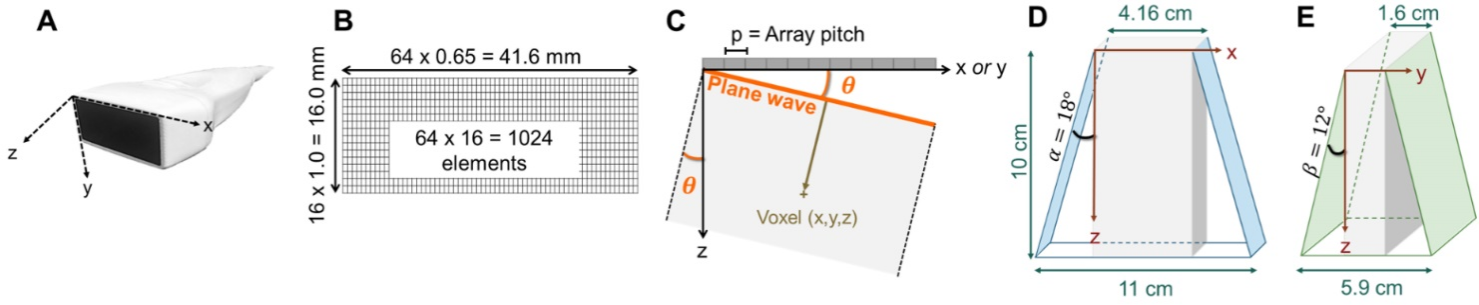
** Representation of the 3D volume extension resulting from plane wave imaging with 64 × 16 -element array**: (**A**) photograph of the array and axis definition: *x* corresponding to the largest dimension of the array (azimuth), *y*, to the shortest, and *z*, to the propagation depth, (**B**) 1024-element array geometry, (**C**) geometrical definition of the plane wave angle, (**D**) azimuthal extension at imaging depth 10 cm, where α defines the azimuthal steering angle, (**E**) elevational extension at imaging depth 10 cm, where β defines the elevational steering angle. The 1024 channel, 4x256 imaging system configuration is shown in Fig 14.

Based on imaging and therapeutic mode simulations, λ and 1.5 λ imaging pitch were chosen for the azimuthal and elevation axes, respectively, for a 41.6 mm aperture in azimuth and 16 mm aperture in elevation (Figure 3B). In order to fully characterize the field, we then evaluated the 3D normalized pressure field resulting from a transmission by the 2D array at 1.3 MHz for a focused wave (Figure S3) and by a plane wave (Figure S4). The results demonstrate the flexibility achieved by this combination of the 2D array and 1024 channel system in that instantaneous insonation of a localized region of interest or broad plane are feasible providing flexibility for treatment protocols.

### Passive acoustic mapping

The array designs (Figure S5 A1-D1) were also evaluated for 3D passive acoustic mapping in comparison with a commercial 3.5 MHz, 32×32 array 6,58. The angular spectrum method for passive acoustic mapping (AS-PAM) 50 as defined in the Methods section probed the sensitivity of the array to microbubble oscillation as a function of position (Figure S5 A2-D2). The projection on each axis provided a basis for comparison (Figure S5 A3-D3). We quantified the full width at half maximum (FWHM) and computed the root sum square (RSS) of the FWHM in the three dimensions (Figure S5 E). The 64×16 configuration offered the best compromise between FWHM, with the smallest RSS of 6.99, far below the RSS of 25.73 obtained with the commercial 2D array. This confirms 3D PAM as a potential application for the array and system.

### Steering limits

With this array design, each element of the matrix is driven independently and 2D plane waves are generated by applying delays to all elements. Thus, each transmit/receive event uses the entire aperture. The steered 2D plane waves generate volumes that are coherently summed. One challenge lies in finding the optimal number of plane waves and the steering limits. The steering-angle limit is governed by the appearance of grating lobes in the image, itself highly dependent on the pitch size *p*. When the steering angle 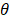 is defined as in Figure 3C, we know that the first one-way grating lobe 59,60 appears at:


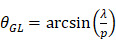

(1)

The proposed maximum steering angle can then be expressed as:


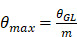

(2)

Where *m* denotes the grating lobe multiplier, a constant reflecting the level of grating suppression. Considering the small angles approximation, 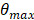 becomes:


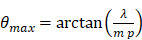

(3)

Empirically, we chose m = 3, which gives a ±18° steering limit in azimuth (where pitch ~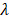), and a ±12° steering limit in elevation (where pitch ~1.5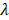). This arbitrary value for m was determined by assuming a -60 dB threshold in the grating lobe appearance at different depths in the image. Azimuthal steering angles are noted as 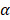, and elevation steering angles as 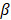 (Figure 3D and 3E, respectively). Each transmitted plane wave is described as a 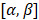 set. At this point, because of the array element asymmetry, we made the choice of fixing one of the above to 0° when the other is non-zero. This is equivalent to assuming that the transmission rotates around one axis, either *x* or *y*, at a time. The resulting steering angle limits provide a field-of-view of 11 cm in azimuth and ~6 cm in elevation at an imaging depth of 10 cm (Figure 3D-E) and ~6 cm in azimuth and ~3 cm in elevation at a depth of 3 cm.

### Acoustic characterization

**Figure 4:**
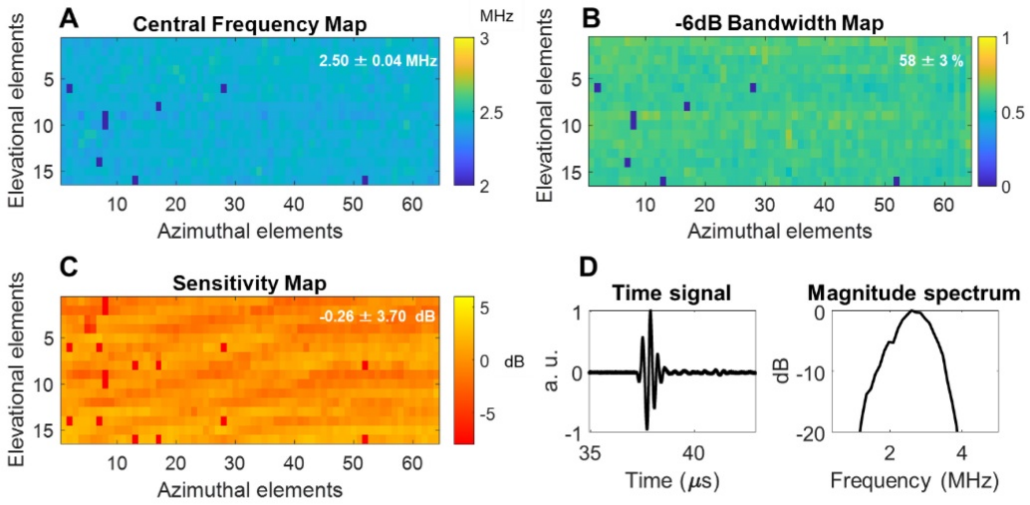
** Acoustic parameters of the 64 × 16 array pulse-echo measurement on a flat crystal**: (**A**) center frequency map, (**B**) -6 dB bandwidth, (**C**) normalized sensitivity, (**D**) one-way time and frequency impulse responses were measured by placing a hydrophone at the natural focus and maximizing the received signal. Locally aberrant elements are dead elements that were not accounted for in the mean and standard deviation computations. a.u. = arbitrary units.

The array was fabricated by Vermon SA (Tours, France) and the acoustic properties were characterized in pulse-echo measurements as detailed in the Methods section. The mean of the center frequency was 2.5 MHz with a standard deviation of 0.04 MHz and a mean fractional bandwidth of ~58 ± 3% (Figure 4A-B). Additionally, a 1-way hydrophone measurement indicated a center frequency of 2.55 MHz and -6 dB bandwidth of ~60% (Figure 4D). The normalized element sensitivity variation averaged over the array was 0.26 ± 3.7 dB (Figure 4C).

### Spatial resolution: azimuth vs. elevation

**Figure 5:**
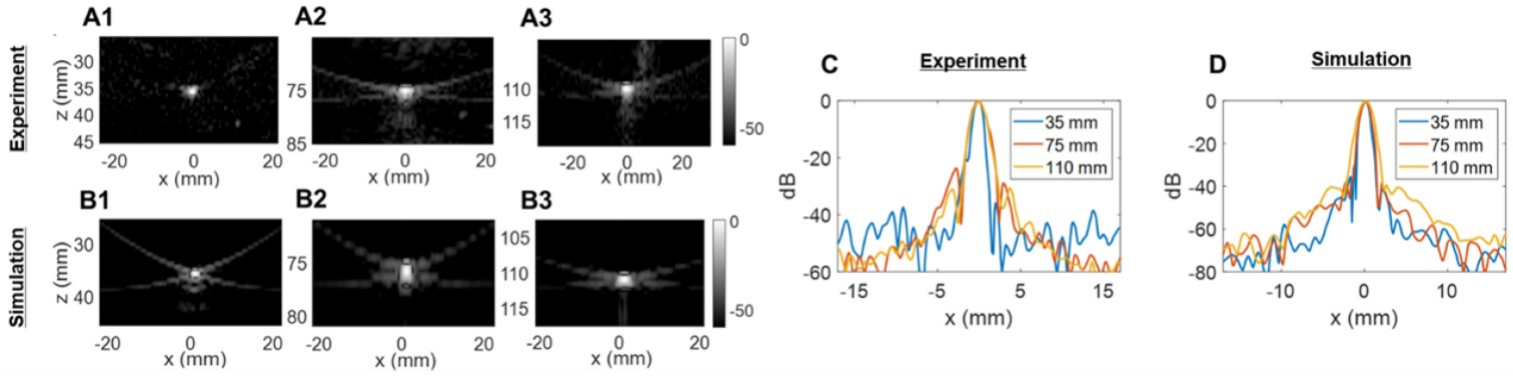
** Two-way point spread function (PSF) estimation in the azimuthal dimension**: (**A**) experimental PSFs obtained in the central azimuthal plane using a tungsten wire, (**B**) Verasonics (VSX) simulated PSFs using the central linear array (single column of the 2D transducer). Three different depths were tested in (A) and (B): 35 mm, 75 mm, and 110 mm. (**C**) Comparison of experimental lateral resolution of the PSFs. (**D**) Comparison of simulated lateral resolution of the PSFs.

**Figure 6:**
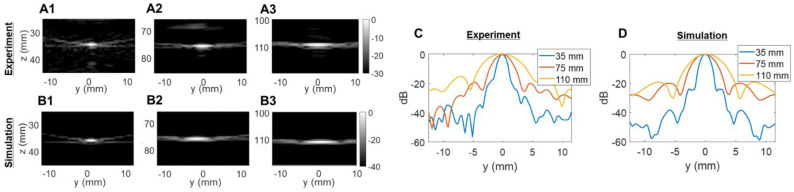
** Two-way point spread function (PSF) estimations in the elevational dimension**: (**A**) experimental PSFs obtained in the central elevational plane using a 25-µm tungsten wire, (**B**) PSFs obtained in the central elevational plane with simulated 2D array. Three different depths were tested in (A) and (B): 35 mm, 75 mm, and 110 mm. Comparison of lateral resolution of the PSFs: (**C**) experiment; (**D**) simulation.

We then assessed the potential imaging quality by evaluating the lab-acquired azimuthal and elevational PSFs for plane wave transmissions and compared these to the expected performance by simulation (Figure 5 and Figure 6). Here, we compared simulated and experimental results for targets located at 3 and 10 cm in depth. The theoretical lateral resolution is given by the diffraction spot size:


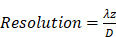

(4)

where λ is the imaging wavelength, 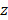 is the imaging depth and *D* is the aperture length. Thus, we expect the PSF to degrade with depth.

Plane waves were chosen to reduce the set of transmissions needed and image the entire volume with the full 1024-element array. Steering limits, determined earlier, set up the angular aperture of plane waves in both directions. To obtain equivalence between cylindrical and plane wave focusing, one needs to transmit at least *N_PW_* angles 61,


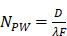

(5)

where F is the f-number defined as the ratio between the imaging depth and the aperture length. Due to our objective of deep imaging, we chose an f-number of 1.5, which gives a plane wave count of 45 in azimuth and 17 in elevation. Thus, as detailed in the Steering limits section and for all data presented in the paper, 45 plane waves in [-18°, 18°] were transmitted in azimuth and 17 plane waves in [-12°,12°] in elevation.

We compared experimental and simulated capabilities of our array both in azimuth (64 elements, λ-pitch) and in elevation (16 elements, ~1.5λ-pitch). To obtain the closest estimation of our prototype design behavior, simulated radiofrequency (RF) signals were obtained by defining the 2D array with the Verasonics simulation tool (VSX). Experimental signals were measured on a 25-µm tungsten wire in a water tank. All data were beamformed with our GPU-based parallel processing algorithm.

The experimentally determined azimuthal resolution at -6 dB was 1.3, 1.8 and 1.9 mm at a 35-, 75- and 110-mm depth, respectively (Figure 5C). The simulation-determined azimuthal -6 dB resolution was 1.1, 1.2 and 1.7 mm (Figure 5D). As we expected, experimental PSFs diverge from simulation results with an error margin below 20%. Similar conclusions can be drawn from the elevation array simulations and experiments where an experimental -6 dB resolution of 2.0, 4.1 and 5.6 mm at 35-, 75- and 110-mm depth, respectively, (Figure 6C) was achieved. The -6 dB simulated resolution was 1.9, 3.9 and 5.5 mm for the 35-, 75- and 110-mm depth, respectively (Figure 6D).

As expected from Equation (4), the resolution in azimuth is ½ that in elevation.

## Extended 3D B-mode imaging

Overall, the 2D array we present here is driven as a stack of 64-element linear arrays steerable up to 18° in azimuth, and as a stack of 16-element linear arrays steerable up to 12° in elevation.

### Importance of elevational steering

**Figure 7:**
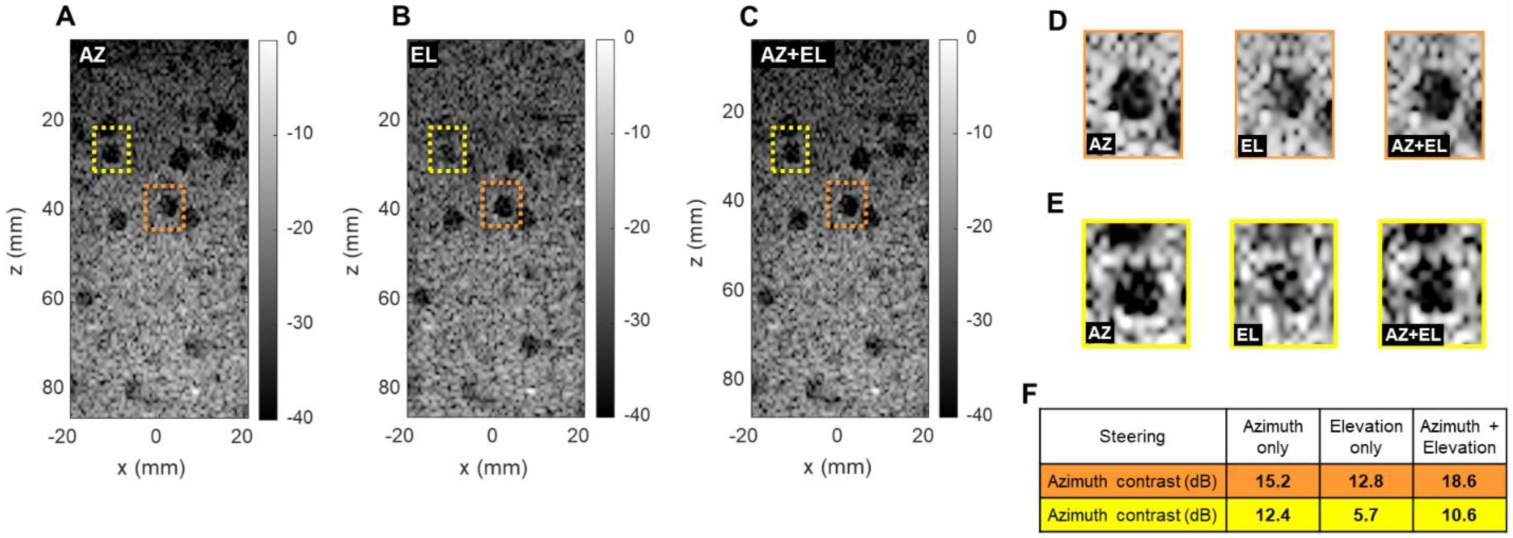
** Effect of azimuthal and elevational steering on spherical cyst contrast (CIRS 050 3D phantom)**. Plane wave B-mode images in x, z plane with (**A**) azimuthal steering (AZ), (**B**) elevational steering (EL), (**C**) azimuthal and elevational steering compounded (AZ+EL). (**D**) Enlarged display of orange cysts (center line in azimuth). (**E**) Enlarged display of yellow cysts (off center). (**F**) Contrast ratio computation in two different phantom regions.

To evaluate the ability to detect small cysts through combinations of azimuthal and elevational steering, we evaluated the detection of spherical 5 mm diameter anechoic cysts in the central (*x*, *z*) plane using a commercially available CIRS 050 spherical cyst phantom (CIRS Inc., Norfolk, VA). We compared three steering configurations: azimuthal steering only, elevational steering only, and a combination of the two. For each case, we selected two different cysts in which we computed the contrast ratio (CR) in dB as follows:


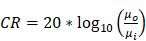

(6)

where *µ_i_* and *µ_0_* respectively are the mean image intensity inside and outside the cyst. The results presented in Figure 7 show two interesting effects. As expected, in-plane (azimuth) only steering (Figure 7A) increases contrast compared with out-of-plane (elevation) steering only (Figure 7B). When both steering capabilities (Figure 7C) are combined, the contrast enhances, which proves that adding elevation steering to the volume beamforming contributes to better in-plane image quality. This effect was observed among all cysts, whether on center line (Figure 7D) or off center (Figure 7E). For cysts that are off center in the azimuthal or elevational planes (yellow in Figure 7) steering in both planes continues to improve the minimum contrast achieved with steering in only one of the two planes (Figure 7F). Overall, this comparison further underlines the importance of elevational steering in small structure detection which is of crucial importance when monitoring therapeutic effects *in vivo*.

### *In vitro* results

We probed the B-mode imaging capabilities of the array on a commercial 3D phantom (ATS 560H). A total of 62 2D ultrasound plane waves were generated by steering 45 angles in [-18°, +18°] in azimuth and 17 angles in [-12°, +12°] in elevation using a PRF of 4 kHz and a maximum depth of 16 cm. We selected two regions of interest in the phantom: the first region had cylindrical cysts and an oblong spherical egg of different echogenicity, while the second region had a network of reflecting pins.

**Figure 8:**
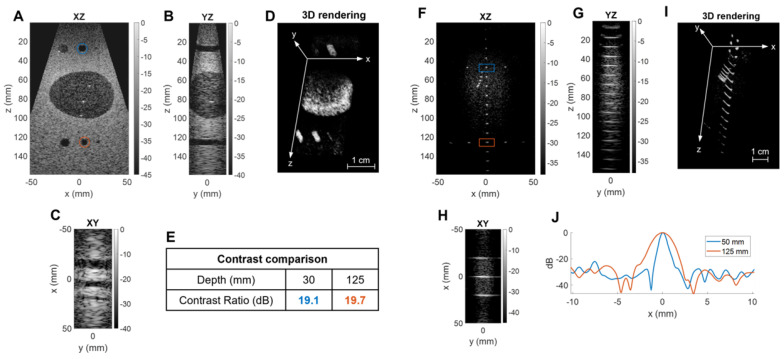
** Three-dimensional B-mode image of a commercial phantom with inclusions of different echogenicity:** Egg and cylinder phantom: (**A**) XZ central slice, (**B**) YZ central slice, (**C**) XY slice, (**D**) 3D rendering with reversed grayscale, (**E**) contrast comparison at two depths. Reflecting wires in speckle: (**F**) XZ central slice, (**G**) YZ central slice, (**H**) XY slice, (**I**) 3D rendering, (**J**) lateral resolution comparison at two depths.

The B-mode images presented in Figure 8 show the capability of the system to map 3D shapes (egg, cylinders, wires) for depths to 15 cm with a wide field-of-view. To evaluate the loss of image quality with depth, we compared both the contrast ratio and resolution at shallow (30 and 50 mm) and larger (125 mm) depths. Contrast ratio, computed in parallel cylinders with Equation (6), yielded 19.1 and 19.7 dB at 30 and 125 mm respectively (Figure 8E). Resolution was estimated with lateral cross-sections of the image intensity and resulted in a -6 dB azimuthal resolution of 0.8 and 2.1 mm at 50- and 125-mm depths, respectively (Figure 8J). As expected by the diffraction laws, the resolution degraded with depth but the contrast remained at the same level.

### *In vivo* results

**Figure 9:**
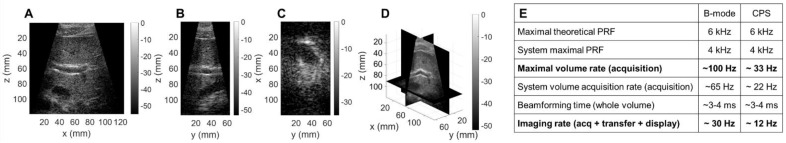
** Three-dimensional B-mode liver image of a healthy volunteer:** (**A**) XZ central slice, (**B**) YZ central slice, (**C**) XY slice, (**D**) 3D rendering of the slice positions. (**E**) Table of volume and imaging rates for *in vivo* imaging of a 13×12×6cm^3^ volume, with a λ^3^ voxel size.

We performed a first assessment of our system in a typical clinical application with *in vivo* liver imaging of a healthy volunteer. We used the same sequence as previously: 45 plane waves steered in [-18°, +18°] in azimuth and 17 in [-12°, +12°] in elevation with a 4 kHz PRF, and an imaging depth of 13 cm. Due to the ability to steer the beam in each dimension, the size of the volume acquired can exceed that of previously reported phased arrays (Figure 9A-C). In one practical example shown here, the operator can select planes of interest and navigate through a 13 cm × 12 cm × 6 cm volume with a λ^3^ voxel (Figure 9D) in real-time. For this configuration (Figure 9E) a *volume (acquisition) rate* up to 100 Hz can be achieved (limited to 60 Hz by the current operating system). A volumetric *image display rate* of ~30 Hz was achieved in B mode.

## Ultrasound-guided therapy

For improved contrast in microbubble imaging, a contrast pulse sequence (CPS) protocol was performed with a transmission frequency of 1.5 MHz (vs 2.5 MHz for B mode) by transmitting three pulses (1/2, -1, 1/2) and coherently summing the echoes. This reduced the *volume rate* by a factor of three to 22 Hz (Figure 9E) and therefore a volumetric *image display rate* of 12 Hz was achieved in CPS mode.

### *In vitro* therapeutic assessment

**Figure 10:**
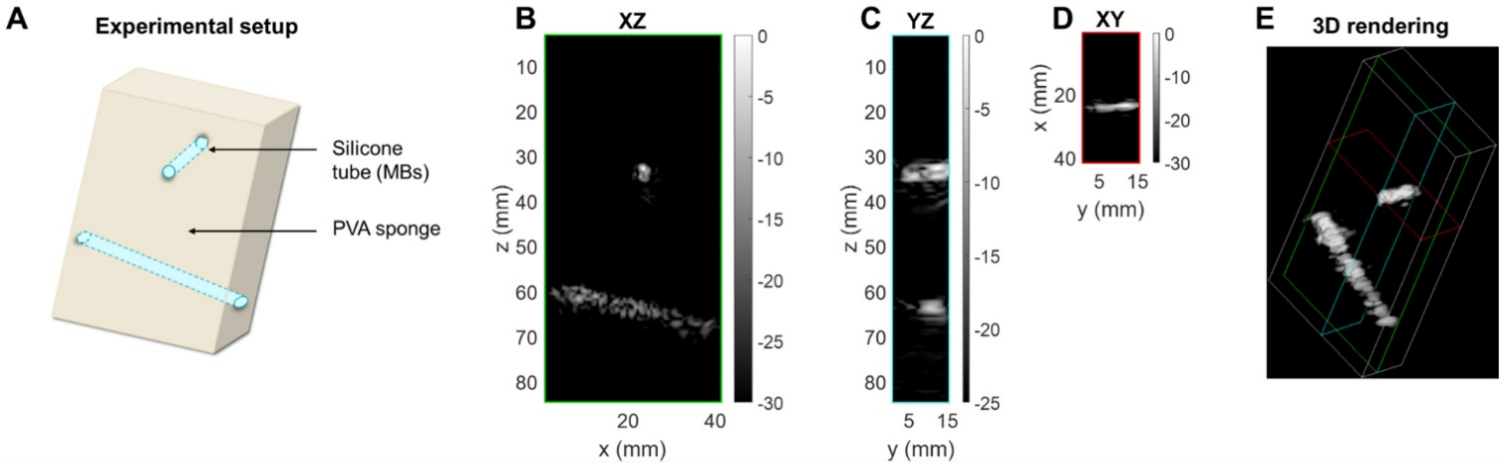
** 3D contrast imaging on a phantom setup.** (**A**) Sketch of the experimental setup: silicone tubes for microbubble (MB) folw imaging are inserted orthogonally to each other in a polyvinyl alcohol (PVA) sponge. Slices of the CPS volume: (**B**) azimuthal XZ, (**C**) elevational YZ, (**D**) coronal XY. (**E**) 3D rendering of the CPS volume. Green, blue and red colored frames indicate the position of the slices respectively displayed in (**B**), (**C**), (**D**).

To assess the performance of the array for CPS and therapy applications, we utilized a custom flow phantom consisting of a polyvinyl alcohol (PVA) sponge with embedded silicone tubes (Figure 10A). Compared to B-mode imaging at the same time after injection, CPS demonstrated a gain in contrast up to 12 dB as well as a suppression of reflection from the silicone tubes which contained the microbubbles. Thus, we imaged the non-linear oscillation of microbubbles during their propagation in the tubes (see Methods section). The three cartesian planes, combined with a 3D rendering offer a true representation of the structures in the *in vitro* medium (Figure 10B-E). The width of the tube in which the microbubbles flow was estimated by measuring the FWHM of the peaks of the CPS signal as 2.8 mm and 4.3 mm at a 35- and 65-mm depth which compares well with the inner diameter of the tube (3 mm).

**Figure 11:**
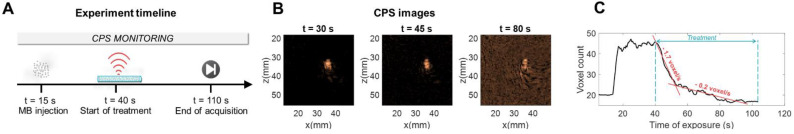
** Therapeutic sequence.** (**A**) Experimental timeline including CPS monitoring. (**B**) CPS images at different time points of exposure. (**C**) Higher intensity pixel count in the central azimuthal plane over time.

After collection of the CPS data, the therapeutic sequence using the same 2D array was applied, as described in the Methods section, for more than one minute while focusing the beam along the upper tube at around a 35-mm depth (Figure 11A). Volumetric acquisition provided the opportunity to quantify voxels of highest intensities. In the vicinity of the upper tube, the voxel intensity in the CPS image was normalized by the maximum intensity over time in this region. Different time points in Figure 11B show the change in the image intensity and speckle with exposure. To provide a quantitative assessment, voxels with intensities above a -30 dB threshold were counted. With the microbubble injection, the count rose instantaneously and remained constant during CPS monitoring (Figure 11C). As soon as the therapeutic sequence was launched, the count decreased sharply at a rate of ~ 2 voxels per second, slowing after 25 seconds.

**Figure 12:**
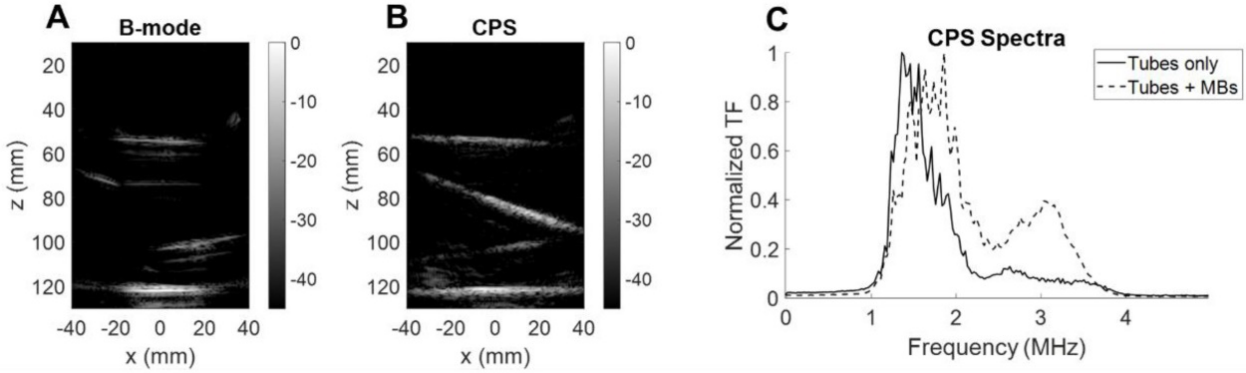
** Microbubble monitoring.** (**A**) B-mode image of silicone tubes in water with microbubbles flowing. (**B**) CPS image of silicone tubes in water with microbubbles flowing. (**C**) Fourier spectra of CPS signal with and without microbubble flow.

To ensure that the microbubbles were oscillating nonlinearly, we quantified the backscattered signal spectrum for microbubbles inside a purpose-built water bath phantom. We compared the CPS results with and without microbubble flow (Figure 12). First, the B-mode image (Figure 12A) highlights the tube interfaces whereas the CPS image (Figure 12B) displayed only microbubbles flowing. When microbubbles were flowing, the spectral differences were evident with a harmonic spectral peak above 3 MHz in the CPS spectrum (Figure 12C). This confirmed the quantification of the nonlinear oscillations with the single integrated array.

### *Ex vivo* imaging of microbubbles

**Figure 13:**
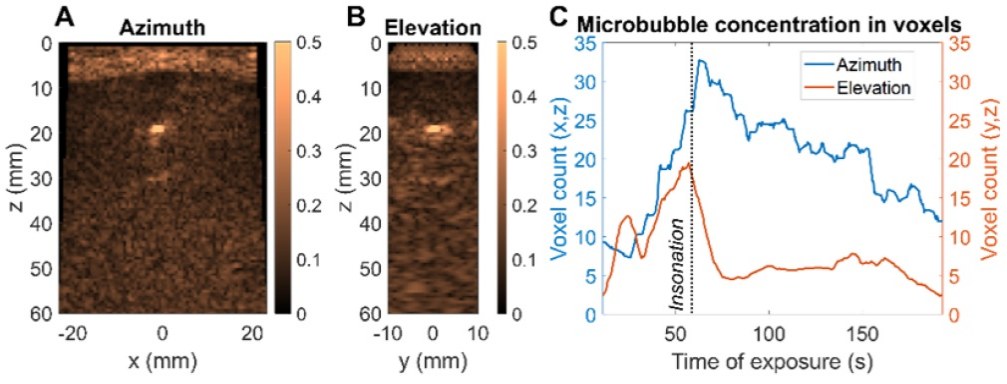
***Ex vivo* microbubble imaging in a mini-pig bone fracture model.** (**A**) CPS image of central azimuth (x,z) plane (**B**) CPS image of central elevation (y,z) plane (**C**) Evolution of microbubble distribution through higher intensity voxel count over time - comparison of azimuth and elevation plane.

Finally, we evaluated a therapeutic protocol in a mini-pig model of nonhealing fracture *ex vivo.* 100 µL of Definity microbubbles, diluted 20 times, were injected. The therapeutic sequence described in the Methods was used to insonify the microbubbles and the image amplitude was assessed in two planes simultaneously (Figure 13A-B). The nonhealing fracture model or condition is particularly challenging for delivery and guidance of microbubble-based therapy as the highly reflective bone limits the ultrasound penetration and image guidance (Figure 13C). The decrease in CPS intensity was tracked in each plane as the microbubbles were destroyed. The focused beam was translated along the x-axis (azimuthal direction) to address the region of interest. We confirmed the ability to steer the imaged planes to the region of interest and to observe the differential reduction in image intensity in the orthogonal planes. This demonstrates the key feature of monitoring the microbubble-based intensity and the resulting rate of signal reduction for individual planes of interest. As expected, microbubbles are destroyed more rapidly in regions with a lower microbubble density and lower image intensity (Figure 13C). With the ability to monitor the spectrum and changes in intensity in arbitrary planes, treatment can be optimized.

## Discussion

Our goal was to create a 2D array integrated with a 1024 channel real-time system and provide proof of concept for human imaging and microbubble-based therapy. As compared with commercial 2D arrays which reduce the channel data to ~128 channels using locally integrated micro-beamformers in the probe handle, we explored the use of a custom array with simultaneous acquisition on all 1024 elements, providing the flexibility to explore ultrasound-guided microbubble therapy. First, we noted that the direct connection between the elements and dedicated transmitters may be particularly important for therapeutic use, where longer pulses are required and could potentially cause overheating if the transmitters are embedded in ASICs. The requirements for ultrasound-guided microbubble therapy include: large field-of-view and high contrast, high-volume acquisition rates and narrow bandwidth on transmission with sufficient receive bandwidth. We address each of these here.

We first address the field-of-view, spatial resolution and grating lobes. When limited to 1024 elements in a 2D array, difficult choices must be made as to the pitch in each dimension. By optimizing the number of elements and pitch in the elevation and azimuthal dimensions, we achieved the desired volumetric region of interest. We found that a configuration with 64 elements separated by a λ-pitch in azimuth and 16 elements separated by a 1.5λ-pitch in elevation allowed for microbubble imaging and therapy in 3D. When imaging at 2.5 MHz, for the 64 × 16 array, the grating lobe height remained lower than the 32 × 32 array in azimuth and the 128 × 8 array in elevation. This design creates grating lobes below 40 dB for a region of ±50 mm in either dimension and would likely minimize the impact of grating lobes on the resulting image. For therapy, the off-target intensity was below -40 dB over a range of positions spanning more than 60 mm in each dimension and therefore off-target effects would not be expected. This design thus provided an extended field-of-view to 11 cm in azimuth by 6 cm in elevation at 10 cm in depth, with steering of ±18° in azimuth and ±12° in elevation.

High volume acquisition rates are important for interventional applications where microbubble dynamics must be visualized and contrast agents potentially injected under image guidance. The use of plane waves facilitates ultrafast ultrasound applications. The system is capable of imaging 1024 channels at pulse repetition frequencies up to 4 kHz and implements highly parallelized imaging algorithms thanks to the use of GPUs. The design we are proposing here allows imaging as deep as 20 cm in 3D and in real-time so that the therapeutic treatment can be monitored with a large field-of-view. At a depth of 16 cm in B-mode, we achieved a *volume acquisition rate* of 60 Hz and an* imaging display rate* of 30 Hz. Lateral resolution was 0.8 mm at 5 cm depth and 2.1 mm at 12.5 cm depth. In the example provided, our system was capable of achieving the spatial and temporal parameters required for combined ultrasound imaging in real-time and image-guided therapy. In a clinical context, one could think of offering the physician the option to select a given set of planes, from 1 to 8, to display which could reduce the demands on the real-time capacity of the system. Future work will include 4D Doppler 62 imaging of liver or kidney on healthy volunteers.

After applying cable-based tuning to reduce harmonics in the transmitted signal, the -6dB receive bandwidth was ~58% and was sufficient for the application. There are a number of electrical impedance matching or “tuning” topologies which can be used to optimize bandwidth and sensitivity with a slight increase in system complexity depending on the type of implementation 63, 64. Our probe was optimized for sensitivity at the expense of bandwidth with series inductors in the system side connector. While a broad bandwidth is necessary for harmonic imaging, a slightly reduced bandwidth is beneficial in minimizing extraneous harmonic content in the transmitted signal. Our experience is that tuning is required for integration with the pulsers in this commercial system in order to avoid the transmission of harmonics that prevent high quality microbubble imaging. The integrated array was capable of transmission and reception between 1.3 to 4 MHz, which is required for microbubble oscillation and reception of harmonic components. We probed this ability to detect harmonics through CPS imaging of microbubble nonlinear oscillation in a phantom. In imaging, such frequencies also allowed tissue penetration and CPS imaging to 16 cm. We found that the transducer was capable of differentiating linear and nonlinear oscillation and detecting the harmonic oscillations from microbubbles within a phantom. Therefore, we confirmed that the spectral performance of the transducer and system were sufficient for the application.

We also demonstrated that a rectangular 2D array can have advantages for 3D passive acoustic mapping (PAM) in that this geometry provided superior localization of the microbubble emissions. In the future, half of the array in elevation - a 64 × 8 sub-array - can be used to sonicate the microbubbles, and the other half will passively receive the echoes. The array could monitor acoustic cavitation during the therapeutic sequence 65-67 although with a reduced elevation focusing capability or could monitor cavitation from a second therapeutic array. One limitation is the maximum frequency (~ 4 MHz), which is lower than the 5-10 MHz range used in many PAM configurations; however, the localization is improved.

The technology described here has limitations in peak pressure and time averaged intensity, common to all ultrasound systems. The array temperature should be monitored when transmitting long or high intensity pulses. The bandwidth is also limited: the 1-4MHz range is ideal for microbubble applications and deep imaging but limits higher frequency protocols. In its prototype form, the system is currently cumbersome but this will be reduced in the future. Because we opted for a better resolution in azimuth and a wider field-of-view, the elements of the 2D array are not square, and this prevents us from transmitting plane waves inclined in both azimuthal and elevational direction 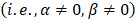.

### Future work

A challenging aspect of ultrasound imaging formation arises when working with heterogeneous and complex media, where aberrations can strongly distort the wavefront. The conventional Delay-And-Sum beamformer then fails to build an accurate image of the medium, and aberration correction techniques should be implemented. The ability to record from and drive each of the 1024 elements of the array, and to perform ultrafast imaging, therefore offers an ideal framework. When microbubbles are injected in the medium, they can act as point sources whose echoes can be isolated. The phase law to correct the echo towards a hyperbolic shape can then be used as a delay on each channel. This will result in better image quality and better focusing capabilities as well 68,69. Similarly, aberration laws can be considered using anatomical information for instance from computed tomography (CT) scans in transcranial applications. Some recent approaches allow fast aberration correction such as the singular value decomposition (SVD) Beamformer 70 will be implemented in future work to improve the coherence between 2D plane waves.

To further extend the transducer aperture, two options can be investigated: further increase the channel count of the system or use a multiplexed array. In a therapeutic application, the power delivered must be considered in the design of any multiplexed system. With larger aperture sizes, a priority would be to decrease the element size in elevation since grating lobes can reach -30 dB and reduce the maximal steering angle. Also, square arrays could improve the symmetry of the resolution in azimuth and elevation directions and allow simultaneous steering in both directions *i.e.*, at 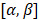 with both angles being non-zero. Such steering capability will result in an isotropic point spread function in the (x,y) plane and thus, an enhanced resolution. This would also enrich the acquisition by illuminating the volume in novel directions, thus improving the coherent compounding between 2D plane waves.

In summary, this work proposes a novel approach for ultrasound imaging and microbubble-based therapy by linking a 2D array with a 1024 channel real-time system that transmit plane waves in the elevational and azimuthal directions. For the first time, 3D ultrasound is used for 3D insonation of microbubbles, with a single array providing the insonation, therapy monitoring, and 3D imaging of the surrounding volume with a large field-of-view at high imaging rates. The work paves the way to extended aperture arrays that will offer access to whole sections of organs and thus enhance the information provided to the clinician at novel levels of time, frequency, and spatial resolution.

## Methods

### Acoustic characterization of the array

To characterize the acoustic properties of each array element, we placed a 90 mm-thick planar crystal, used as the reflector, at 16 mm from the probe. An impulse was transmitted by a given element, and its temporal response was received. All results presented in the paper were obtained by driving the array with a 1024-channel programmable research system (Verasonics Inc, WA, USA). A Fourier transform of the received signal then gave access to the -6 dB bandwidth determination. The center frequency was computed as the arithmetic mean of the upper and lower frequencies in this bandwidth. Finally, sensitivity was evaluated as the ratio between the maximum amplitude received on a given element and the average maximum amplitude over all elements. This process was performed individually for each element of the array (Figure 4). One-way time and frequency impulse responses were measured by placing a hydrophone (HNP-0400, Onda, Sunnyvale, CA) at the natural focus of the array (~ 7 cm) and transmitting a plane wave. The position of the hydrophone was adjusted with a micrometric stage to maximize the received amplitude.

### 3D partial Beamforming implementation

The image formation process, so-called beamforming, consists of computing the image of one voxel in the medium from the knowledge of its pulse-echo response. Therefore, we used the classical Delay-and-sum (DAS) method compensating for the propagation delay in both directions. To do so, we reasonably assume that the arrival time of the plane wave on a voxel corresponds to the propagation time between the orthogonal projection of this voxel on the front and the actual voxel (Figure 3C). Thus, a given plane wave, steered at *α* in azimuth and β in elevation, reaches the voxel (*x*,*y*,*z*) at the time:


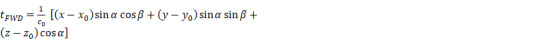

(7)

Where (*x*_0_, *y*_0_, *z*_0_) defines the origin of the wavefront. This point can be seen as the first firing element when transmitting the plane wave. Thus, it is different for each set (*α, β*), and preliminarily determined. The time to travel back to the *i*-th element of the array, of coordinates (*x*_i_, *y*_i_, *z*), is given by:


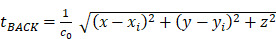

(8)

This two-way propagation delay compensation is actually equivalent to performing both transmit and receive focusing. As discussed before, for a single plane wave transmission (*α, β*), one retrieves a single beamformed volume. To improve resolution and Signal-to-Noise Ratio, multiple angles were transmitted, and the resulting volumes coherently compounded.

**Figure 14:**
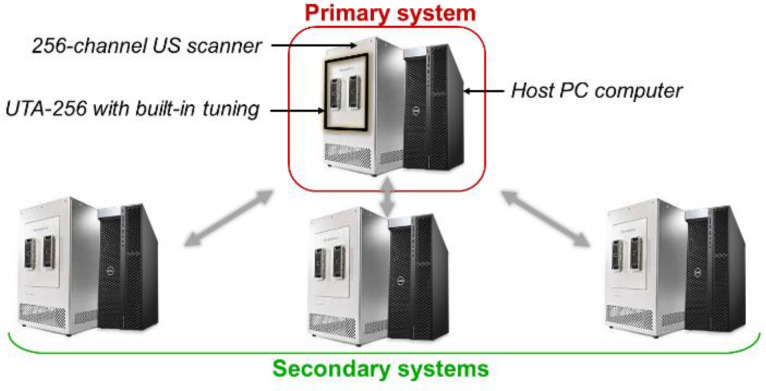
** Global 1024-channel system architecture**. Four 256 channel systems are combined. Each is composed of an ultrasound scanner and a host computer and equipped with a UTA 256 adaptor corresponding to one fourth of the array connectors. Secondary systems are defined and synchronized to an elected primary system.

Methods used to beamform the matrix array data are similar to classical DAS beamforming 51. However, the amount of data to process is strongly increased by the addition of a third spatial dimension. The rise of ultrafast ultrasound imaging was mostly permitted thanks to the development of GPUs that drastically reduced the computation times. Similarly, here, we designed CUDA algorithms to run the beamforming processes on high-end GPUs (Titan RTX, Nvidia, Santa Clara, CA). As mentioned earlier, our array is driven by a 1024-channel ultrasound scanner. This scanner is the combination of four 256-channel scanners, each of them being controlled by its own host PC computer (Figure 14). Impedance matching via 10 µH inductors was added on each channel between the element connectors on the array and the transmitters on the scanners in order to reduce reflections. A partial beamforming approach was set up to optimize the use of available computing resources. One of the systems was selected to be the *primary* system, placing it in charge of the synchronization of the processes and image display, while the three others were assigned to be *secondary* systems. The ultrasound transmit sequence was designed independently and, thanks to the *primary* host computer clock, transmit/receive events occurred simultaneously on each system as if it was a single 1024-element system. Instead of transferring 1024 radiofrequency (RF) signals to the master computer and beamforming all at once, each system, *primary* and *secondary*, performed its own beamforming of the raw 256-channel RF data. The three *secondary* complex quadrature signal (IQ) volumes were then transferred to the master, which compounded them with its own IQ to provide the final beamformed volume.

### Therapeutic setup

In the contrast imaging experiments, a polyvinyl alcohol (PVA) sponge was used to mimic biological tissues since it produces typical speckle patterns. The sponge was preliminarily soaked in degassed water. Silicone tubes - of 5 mm outer diameter and 3 mm inner diameter - were inserted in the sponge to provide a contained path for the microbubbles. The experiment model used here consists in injecting microbubbles into the PVA sponge and monitoring their appearance and flow. Phospholipid microbubbles of ~ 10 µm diameter were prepared in the laboratory with similar composition as clinical contrast agents. They were diluted to obtain a ~ 2×10^6^ microbubbles/mL concentration. Microbubbles were continuously injected into the silicone tube during the ultrasound acquisition at a rate of 0.5 mL/min. A therapeutic sequence was implemented at a duty cycle of ~9% to specifically focus on the microbubbles in the medium. The transmitted signal consisted of 60-cycle bursts at 1.3 MHz. The emission was cylindrically focused at a 35-mm depth, at the location of the first silicone tubes. Thus, the maximum intensity was located along a line parallel to the y-axis. Transmitted voltage was calibrated to induce a 700 kPa pressure, and thus achieve a mechanical index (MI) of 0.6 at the location of the treatment.

**Figure 15:**
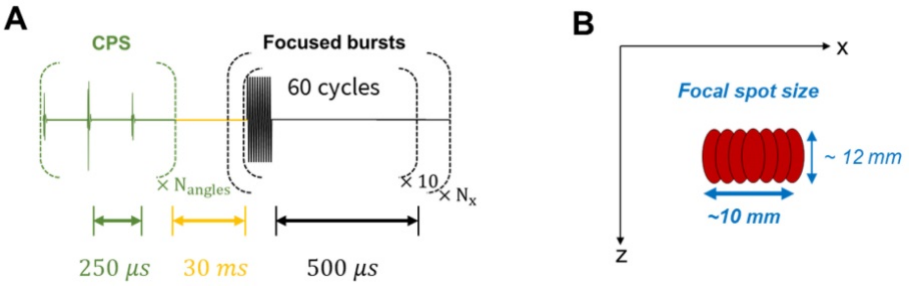
** Therapeutic sequence description**. (**A**) Transmitted signal scheme displaying interleaving delays. (**B**) Final focal spot size representation after successive steered focused beams.

The therapeutic sequence was applied alternatively with the CPS imaging sequence to continuously monitor the effect of the focused beam on the microbubbles as described in Figure 15. One therapeutic sequence is composed of a repetition of 10 bursts, each comprising 60 half-cycles and successively applied at N_x_ = 10 steering positions around the central location. Steering positions were translated by 1 mm alternatively around the center to reach a ~10×12 mm target region. These parameters were set for the *in vitro* experimental proof-of-concept and are specific to the application and the target tissue.

### Passive acoustic mapping (PAM)

Considering the three designs in Figure 1 with equivalent surface area, as well as an available 3.5 MHz 32×32 commercial array (6,58) manufactured by Vermon (Tours, France), we used the homogeneous angular spectrum method for passive acoustic mapping (AS-PAM) to evaluate the performance 50. From simulated RF data, we built AS-PAM reconstructions of microbubble emissions from a point source placed at 50 mm depth. For a source placed at 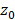 depth, the transfer function is first defined at each frequency ω as the forward propagation spectral operator H 71:


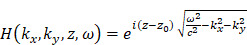

(9)

RF signals are simulated with MATLAB (Mathworks, Cambridge, MA) and the 3D PAM intensity is computed as:


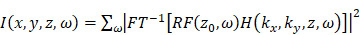

(10)

After normalizing by the maximum value over the entire volume and setting a detection threshold of 0.5, we evaluated the 3D AS-PAM field.

## Supplementary Material

Supplementary figures.

## Figures and Tables

**Table 1:** Goal specifications for the two-dimensional array prototype design

	Imaging	Therapy
Transmission center frequency	~ 2 MHz	~1.3 MHz
Number of array elements	1024	1024
Receive frequency range	2-6 MHz	1.3-4 MHz
Spatial resolution (F# = 1.5)	0.5-1 mm	~1 mm
Target imaging depth range	3-10 cm	3-10 cm
Temporal resolution	Based on depth and scanner PRF capabilities	Complete treatment within 5 minutes
Field of view	5 × 10 cm at 10 cm depth	3 cm × 6 cm
