# Supplementary material for: A theranostic 3D ultrasound imaging system for high resolution image-guided therapy
Source: Theranostics. 2022 Jun 27;12(11):4949–64. doi: 10.7150/thno.71221 (PMC9274734; doi:10.7150/thno.71221)
Supplement: Supplementary file 1 — Supplementary figures. [file thnov12p4949s1.pdf]

A theragnostic 3D ultrasound imaging system for high resolution image-guided therapies

[1]Hanna Bendjador, [1]Josquin Foiret, [2]Robert Wodnicki, [3]Douglas N Stephens, [4]Zoe Krut, [1]Eun-Yeong Park, [4]Zulma Gazit, [4]Gadi Pelled, [1]Katherine W Ferrara

Supplementary Material

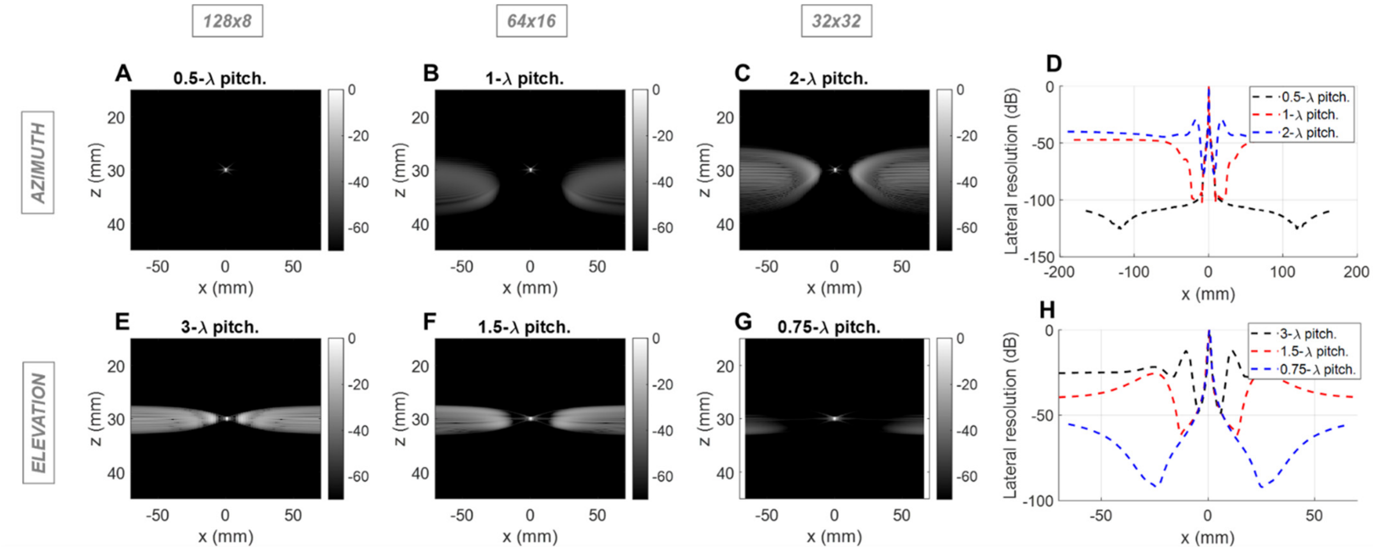

Figure S1: Field II simulations of 2.5 MHz pulse-echo (2-way) point spread functions (PSFs) at 3 cm depth for three array configurations with constant aperture size. Simulations of the azimuthal array: B-mode images for (**A**) 0.5λ – pitch array with 128 elements, (**B**) λ pitch array with 64 elements, and (**C**) 2λ – pitch with 32 elements, (**D**) Lateral resolution (cross-sections) of the PSFs. Simulation of the elevational array: B-mode images for (**E**) 3λ – pitch array with 8 elements, (**F**) 1.5λ - pitch array with 16 elements, and (**G**) 0.75λ – pitch with 32 elements, (**H**) Lateral resolution of the PSFs.

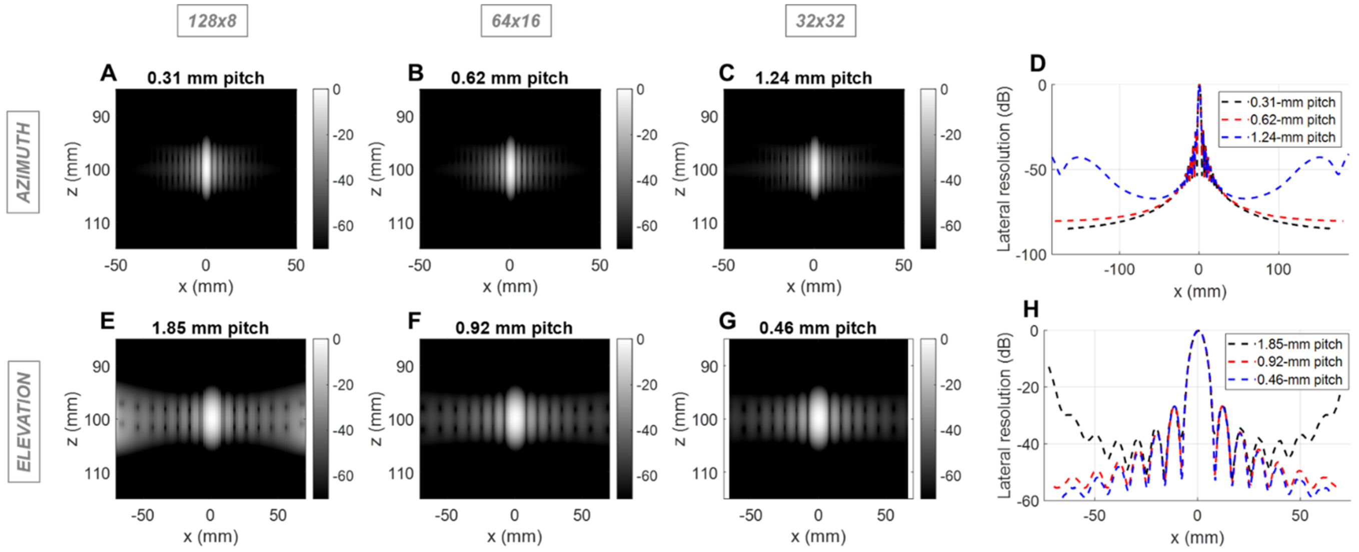

Figure S2: Field II simulations of 1.3 MHz transmitted (1-way) point spread functions (PSFs) at 10 cm depth for three array configurations with constant aperture size. Simulations of the azimuthal array: PSF intensities for (**A**) 0.31 mm pitch array with 128 elements, (**B**) 0.62 mm pitch array with 64 elements, and (**C**) 1.24mm pitch with 32 elements; (**D**) lateral resolution of the PSFs. Simulation of the elevational array: PSF intensities for (**E**) 1.85 mm pitch array with 8 elements, (**F**) 0.92 mm pitch array with 16 elements, and (**G**) 0.46 mm pitch with 32 elements; (**H**) Lateral resolution of the PSFs.

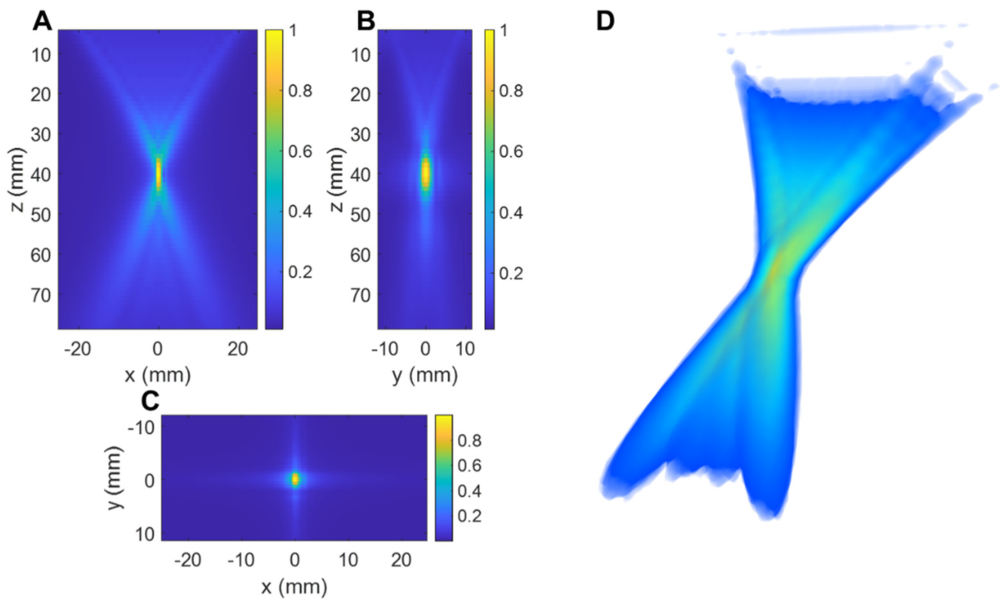

Figure S3: Normalized pressure fields for a simulated focused wave transmitted with the 2D array at 1.3 MHz. (**A**) (x,z)-plane. (**B**) (y,z)-plane. (**C**) (x,y)-plane. (**D**) 3D rendering of the pressure field.

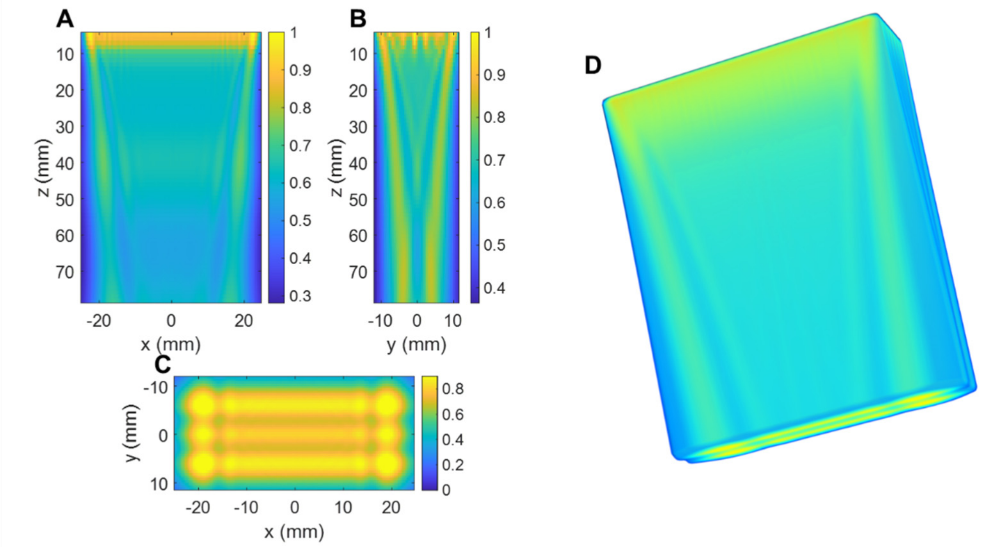

Figure S4: Normalized pressure fields for a simulated plane wave transmitted with the 2D array at 1.3 MHz. (**A**) (x,z)-plane. (**B**) (y,z)-plane. (**C**) (x,y)-plane. (**D**) 3D rendering of the pressure field.

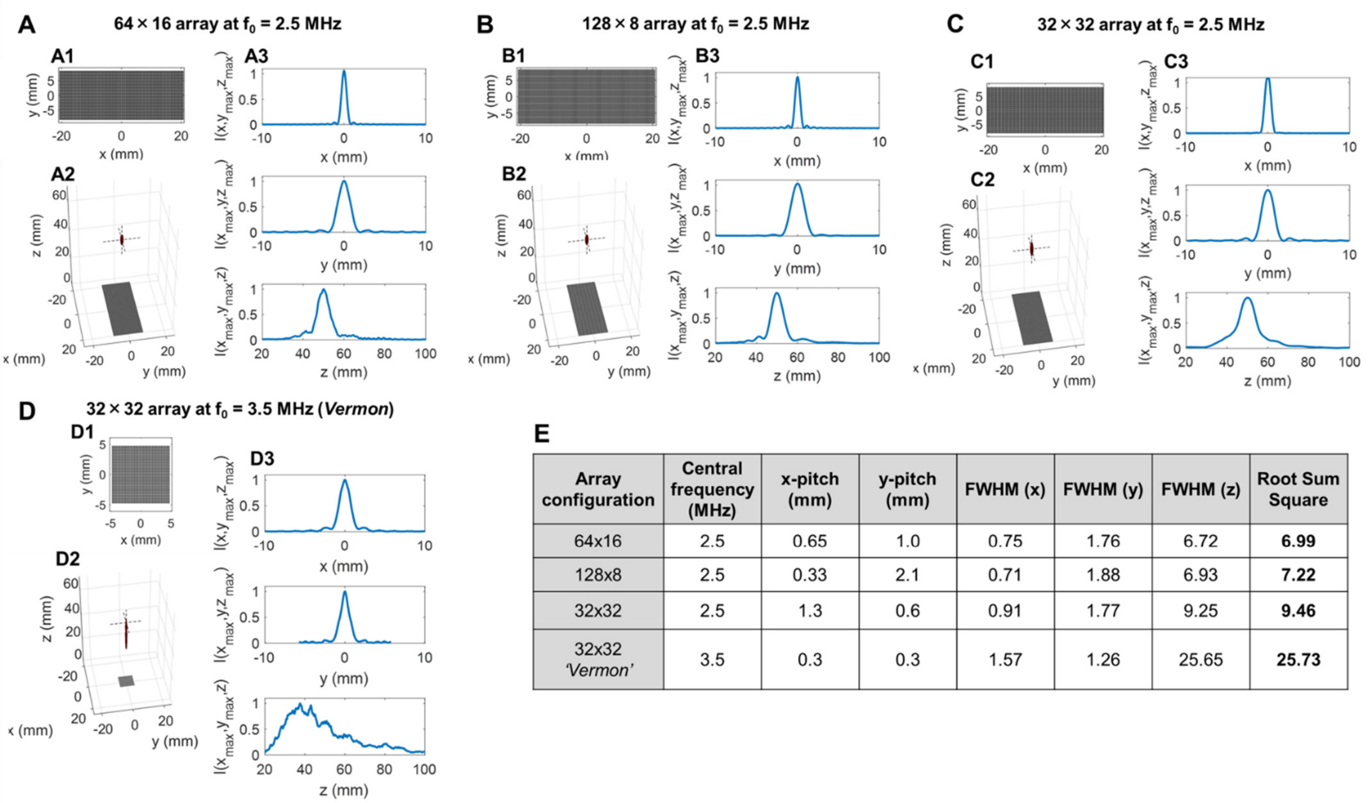

Array configuration	Central frequency (MHz)	x-pitch (mm)	y-pitch (mm)	FWHM (x)	FWHM (y)	FWHM (z)	Root Sum Square
64x16	2.5	0.65	1.0	0.75	1.76	6.72	**6.99**
128x8	2.5	0.33	2.1	0.71	1.88	6.93	**7.22**
32x32	2.5	1.3	0.6	0.91	1.77	9.25	**9.46**
32x32 *'Vermon'*	3.5	0.3	0.3	1.57	1.26	25.65	**25.73**

Figure S5: Spatial resolution obtained with array geometries as applied for passive acoustic mapping. (**A**) 64×16 array with x-pitch = 0.65 mm. y-pitch = 1.0 mm. (**B**) 128×8 array with x-pitch = 0.33 mm. y-pitch = 2.1 mm. (**C**) 32×32 array with x-pitch = 1.3 mm. y-pitch = 0.6 mm. (**D**) 32×32 array with x-pitch = 0.3 mm. y-pitch = 0.3 mm. For each inset: (**1**) array element geometry. (**2**) 3D plot of FWHM of the PAM. (**3**) Projected 3D PAM on x-, y-, and z-axes. (**E**) Table of FWHM results for the four configurations in three dimensions.
